# Iodine status during child development and hearing ability: a systematic review

**DOI:** 10.1017/S0007114522001441

**Published:** 2023-03-14

**Authors:** Mariana Dineva, Amanda Hall, Muqiu Tan, Anna Blaskova, Sarah C. Bath

**Affiliations:** 1 Department of Nutritional Sciences, Faculty of Health and Medical Sciences, University of Surrey, Guildford GU2 7XH, UK; 2 Department of Audiology, School of Life and Health Sciences, Aston University, Birmingham B4 7ET, UK

**Keywords:** Iodine, Hearing, Pregnancy, Children, Cochlea, Auditory development

## Abstract

Iodine, through the thyroid hormones, is required for the development of the auditory cortex and cochlea (the sensory organ for hearing). Deafness is a well-documented feature of endemic cretinism resulting from severe iodine deficiency. However, the range of effects of suboptimal iodine intake during auditory development on the hearing ability of children is less clear. We therefore aimed to systematically review the evidence for the association between iodine exposure (i.e. intake/status/supplementation) during development (i.e. pregnancy and/or childhood) and hearing outcomes in children. We searched PubMed and Embase and identified 330 studies, of which thirteen were included in this review. Only three of the thirteen studies were of low risk of bias or of good quality, this therefore limited our ability to draw firm conclusions. Nine of the studies (69 %) were in children (one RCT, two non-RCT interventions and six cross-sectional studies) and four (31 %) were in pregnant women (one RCT, one cohort study and two case reports). The RCT of iodine supplementation in mildly iodine-deficient pregnant women found no effect on offspring hearing thresholds. However, hearing was a secondary outcome of the trial and not all women were from an iodine-deficient area. Iodine supplementation of severely iodine-deficient children (in both non-RCT interventions) resulted in improved hearing thresholds. Five of six cross-sectional studies (83 %) found that higher iodine status in children was associated with better hearing. The current evidence base for the association between iodine status and hearing outcomes is limited and further good-quality research on this topic is needed.

Iodine, as part of the thyroid hormones, is crucial for brain development^(1)^, and it is now well known that severe iodine deficiency may result in profound neurological impairment and endemic cretinism^(2,3)^. A number of observational studies have shown that even milder forms of iodine deficiency in pregnancy are associated with suboptimal neurodevelopmental outcomes in the offspring, such as reading^(4)^, intelligence quotient (IQ) scores^(4)^, language skills^(5)^ and school performance^(6,7)^; though others have not found such associations^(8–10)^.

Alongside mental deficiency, congenital deafness is another well-documented clinical feature of endemic neurological cretinism observed in areas of severe iodine deficiency and endemic goitre^(3)^. Sufficient iodine intake is required for optimal thyroid function^(11)^; thyroid hormones are involved in auditory development^(12)^ and studies in rodents have demonstrated the role of triiodothyronine (T_3_) at several levels in the auditory system (e.g. outer and middle ear, inner ear, brainstem and brain auditory pathways)^(12)^. T_3_ plays a crucial role particularly in the development of the cochlea (the sensory organ for hearing) in the inner ear^(12–14)^. The development of the human auditory system begins *in utero* with the formation and maturation of the cochlea, but it also continues during early and late childhood when the maturation of the auditory cortex occurs^(15)^. It has been suggested that the period of auditory development that is most sensitive to T_3_ occurs predominantly *in utero*, but developmental events in early postnatal life might also be T_3_-sensitive^(12)^.

Although the association between iodine deficiency and cognition has been widely explored in the context of milder forms of iodine deficiency (i.e. mild-to-moderate), the association between less severe forms of iodine deficiency and hearing in individuals without clinical features of endemic cretinism is relatively underexplored and therefore the more subtle effects of iodine deficiency on hearing ability are unclear. A previous narrative review in 2013 brought attention to this topic and concluded that there were limited number of studies that had investigated the link between iodine deficiency and auditory performance; however, most studies included in the review were suggestive of an association^(16)^. It has been almost a decade since that narrative review was published and the current evidence is unclear and warrants further investigation.

Suboptimal iodine intake is not confined only to iodine deficiency, and it can manifest as iodine excess; both scenarios may be harmful for brain development^(17)^ and therefore the association between iodine nutrition and auditory development should also be explored across the full range of iodine status/intake.

Although most research in mild-to-moderate iodine deficiency is focussed on cognitive outcomes, hearing is also an important outcome, both as a stand-alone effect and as a potential mediator of the association between iodine status and other cognitive outcomes investigated in previous studies. Hearing impairment, especially if unaddressed, can be very damaging for individuals, particularly for children and young people, as well as for the society and the economy^(18)^. Hearing problems can have a negative effect on language development and communication and may adversely affect school performance, cognitive and social skills, and it may also lead to unemployment or underemployment^(18)^. Even mild hearing impairment, which could remain unnoticed, may adversely impact speech and language development in children^(19–21)^. Data from the WHO show that the global yearly cost of unaddressed hearing impairment is 980 billion USA dollars^(18)^.

Considering the role of iodine in auditory development and the great individual and societal impact of hearing impairment, the aim of this study was to systematically review and summarise the evidence for the association between: (i) iodine exposure (intake/status/supplementation) during pregnancy and hearing ability in the offspring and (ii) child iodine exposure and hearing ability in childhood or later in life. Based on the findings, we also aimed to review the knowledge gaps and provide future research directions in this area.

## Methods

We followed the updated Preferred Reporting Items for Systematic Reviews and Meta-Analyses (PRISMA) guidelines^(22)^ in the reporting of this systematic review. The review is registered with the International prospective register of systematic reviews (PROSPERO: CRD42021226223); the study protocol is available online (www.crd.york.ac.UK/prospero).

### Search strategy and eligibility criteria

To identify relevant articles, we searched the PubMed (https://pubmed.ncbi.nlm.nih.gov/) and Embase (https://www.embase.com/) databases from inception through to 1 March 2021, using a combination of search terms (online Supplementary Methods). The search was restricted to studies in humans and in the English language. We also identified studies from the reference lists of relevant publications retrieved from the searches and by consulting experts on the topic.

Our inclusion criteria were based on the following: (i) the target exposure – iodine status/intake (assessed using 24-h urinary iodine excretion, urinary iodine concentration (UIC) and/or iodine-to-creatinine ratio measured in urine samples, or estimated iodine intake from dietary assessment), iodine supplementation (any type, dose and regimen) or use of iodised salt; (ii) the target population – iodine exposure in pregnant women and children < 18 years and (iii) the target outcome – any measures of hearing ability/function (e.g. hearing thresholds, hearing impairment, auditory brainstem response, event-related potentials, auditory processing tests) in the offspring of pregnant women or in children/non-pregnant adults ≤ 65 years (in studies where the exposure was measured in childhood); we included this upper age limit for the outcome to capture whether any effects of iodine exposure during development persist into adulthood. To provide a full account of the available evidence, which, to our knowledge, has not been systematically assessed previously, we included all types of study design (i.e. observational studies (including case reports), non-randomised studies of interventions and randomised controlled trials (RCTs)).

Studies that measured the outcome in older adults (> 65 years) were excluded as the focus of this review was not on age-related hearing impairment. Studies that measured iodine exposure in adults (aged 18 years and over) were also excluded, except when a study included data from both children and adults combined. We also excluded studies in Pendred syndrome patients and/or in individuals with Pendred syndrome symptoms or with any genetic mutations, as the hearing defects in these conditions are not as a result of the target exposure (i.e. suboptimal iodine status/intake) but as a result of genetic defects (e.g. iodide organification deficits). We also excluded studies in individuals with known thyroid disease or thyroid cancer, as well as studies where only thyroid function parameters were measured and data on iodine status/intake or iodine supplementation were not available. Studies in languages other than English, animal studies, *in vitro* studies, unpublished or non-peer reviewed articles (e.g. meeting abstracts, letters), as well as narrative reviews/comment articles/editorials and other systematic reviews and/or meta-analysis were also excluded.

### Study selection and data extraction

After retrieving the records from the database searches, duplicates were removed. The abstracts of the remaining search records and the additional records identified from other sources were screened independently by at least two reviewers (M. D., M. T. and A. B.) using an abstract checklist with the inclusion and exclusion criteria. Following the abstract screening, the full texts of the eligible records were retrieved and reviewed independently by at least two reviewers (M. D., M. T. and A. B.). Records with no abstract were examined at the full-text stage. The reasons for the exclusion of the full-text articles were documented, and the remaining eligible full texts were included in the data-extraction stage. Disagreements between reviewers at any screening stage were resolved through discussion.

Data were extracted independently by two reviewers (M. D. and M. T.) using a piloted data-extraction form; extracted data were checked for discrepancies. The data extracted included: (i) general study details – author, publication year, country and study design; (ii) participant details – overall population group, age range for studies in children, sample size, baseline iodine status/intake; (iii) exposure details for observational studies – iodine status/intake based on the measures of urinary iodine excretion or dietary intake mentioned earlier, iodine supplement use (including type, dose, start and duration) or iodised salt use in the study groups, indicators of iodine intake/status other than urinary iodine concentration or its derivatives (e.g. thyroid volume/goitre rate and thyroglobulin (Tg)); (iv) intervention details for intervention studies – type, dose, start and duration of iodine supplement, placebo/control treatment and (v) hearing outcome details – method of hearing assessment, age at testing, assessor and all results on any measures of hearing at any time point. We presented all available effect measures as reported by the authors of each study because the effect measures for the hearing outcome varied across studies. In instances where data were presented on graphs, we estimated the values (where possible) and reported these. For each study, we specified if there was any unclear or missing information. Where necessary, units for the exposure measures were converted, so that UIC and iodine-to-creatinine ratio were expressed in µg/l and µg/g, respectively.

Extracted data were tabulated, and studies were grouped based on the timing of the iodine exposure (during pregnancy *v*. childhood) and then based on study design (in order of the hierarchy of the included evidence i.e. from RCTs to case reports); within each group, studies were presented in chronological order. We were not able to perform any quantitative synthesis of the evidence (i.e. meta-analyses) because of the scarcity of comparable studies; the included studies had differences in the study design and reported outcomes that meant it was not possible to combine into a meta-analysis.

### Risk of bias and quality assessment

The risk of bias of the included RCTs was assessed using Version 2 of the Cochrane Risk-of-Bias tool (RoB 2)^(23)^. Bias in the RCTs was judged in five domains (randomisation process, deviations from intended interventions, missing outcome data, measurement of the outcome and selection of the reported result) as ‘low’, ‘some concerns’ or ‘high’; based on this, an overall risk-of-bias judgement was also assigned to each study (low, some concerns or high)^(23)^. The risk of bias in the non-randomised studies of interventions was assessed using the Risk Of Bias In Non-randomised Studies – of Interventions (ROBINS-I) tool^(24)^; bias was judged in seven domains and as an overall bias across all domains (low, moderate, serious or critical risk of bias) with a similar methodology to that for RoB 2^(23)^. The quality of the observational studies was judged as good, fair or poor using the Newcastle-Ottawa scale (NOS)^(25)^. The risk-of-bias and the quality assessments were performed by at least one reviewer (M. D. and/or M. T.).

## Results

**Fig. 1.**
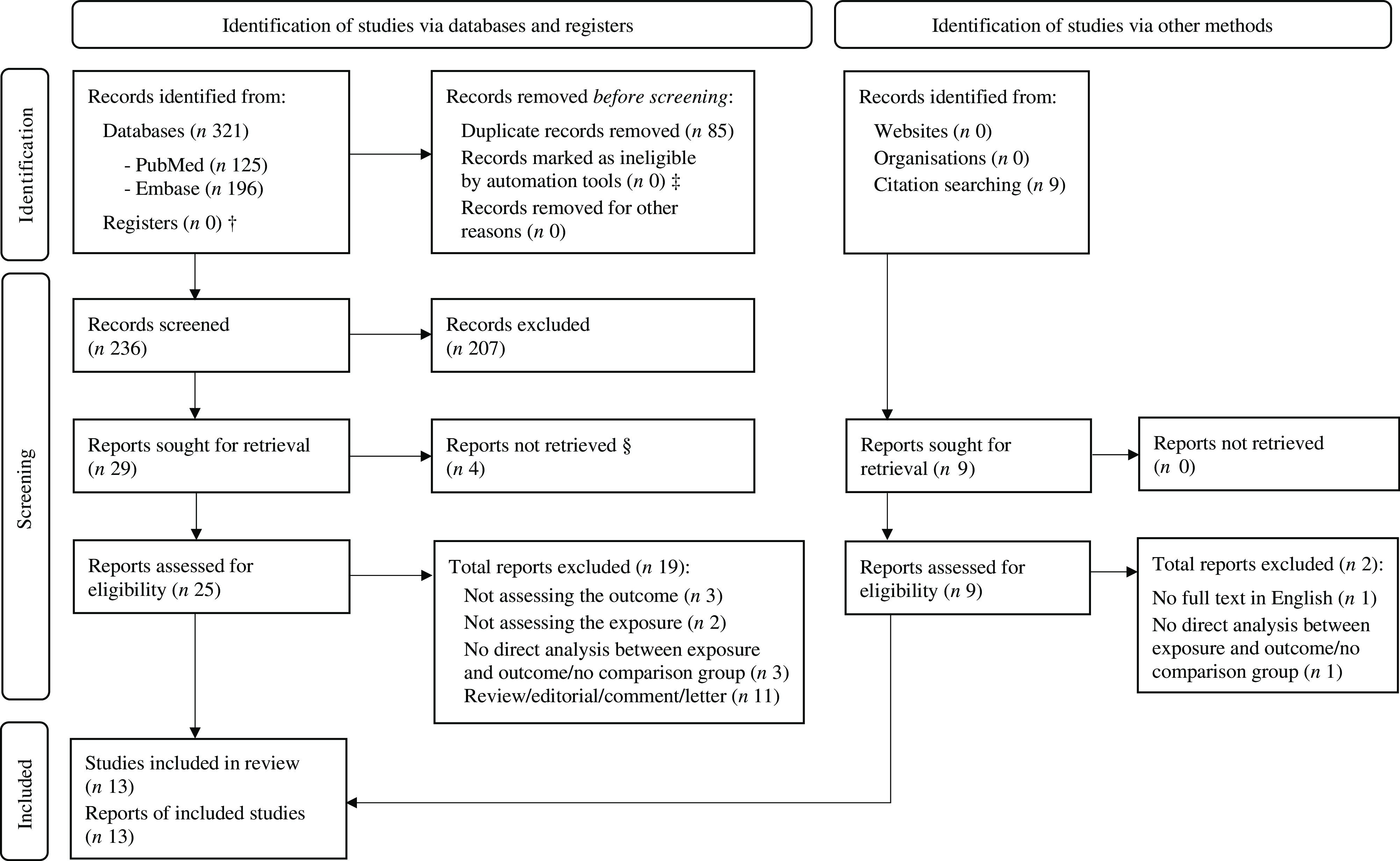
PRISMA flow diagram* of the search results and study selection process. PRISMA, Preferred Reporting Items for Systematic Reviews and Meta-Analyses. *Adapted from Page MJ, McKenzie JE, Bossuyt PM, *et al.* (2021) The PRISMA 2020 statement: An updated guideline for reporting systematic reviews. *BMJ* 372. † No registers were searched as part of this systematic review. ‡ Automation tools were not used in the selection process of this systematic review. § No abstract or full text could be sourced for these reports.

The search yielded a total of 330 records (125 from PubMed and 196 from Embase databases; nine records from the reference lists of relevant publications; Fig. 1). After the duplicates were removed (*n* 85), the abstracts of 245 records were screened. In total, 207 records were excluded after the abstract screening and four reports could not be retrieved, leaving thirty-four full-text reports. A total of twenty-one reports were excluded at full-text screening (with documented reasons; Fig. 1), and the reports of the final thirteen studies were included in this review.

### Characteristics of the included studies

The thirteen studies included were published between 1975 and 2018. The majority (*n* 7, 54 %) were observational (six cross-sectional^(26–31)^ and one cohort study^(7)^) and only four (31 %) were intervention studies (two RCTs^(32,33)^ and two non-randomised studies of interventions^(34,35)^), while the remaining two included studies (15 %) were case reports^(36,37)^.

Studies were from eleven countries: three in the USA^(31,36,37)^, two in Iran^(29,34)^, one each in China^(35)^, Indonesia^(27)^, India and Thailand^(33)^, Benin^(32)^, Zimbabwe^(26)^ and Australia^(7)^. Only two studies were based in Europe: one in France^(28)^ and one in Spain^(30)^. Excluding the two case reports (both based on one case only), total sample size ranged from forty-five to 1252 for the observational studies and from 197 to 234 for the intervention studies.

Most studies investigated the association between iodine exposure during childhood/adolescence (*n* 9, 69 %) and child hearing^(26–32,34,35)^, and only four studies (31 %) investigated maternal iodine exposure during pregnancy in relation to child hearing^(7,33,36,37)^.

### Iodine status in the included studies

In the studies of pregnant women, iodine status was reported as median UIC in two studies^(7,33)^ (range: 99 to 131 µg/l, indicating mild-to-moderate deficiency), and the two case reports in the USA had no measure of iodine intake or status (just use of an iodine supplement that provided excess iodine)^(36,37)^.

In the studies of children and adolescents, iodine status was reported as mean/median UIC in five studies^(28,30–32,34)^ (range: 19 to 181 µg/l) and as mean/median iodine-to-creatinine ratio in six studies^(26,27,29–31,35)^ (range: 16 to 130 µg/g); two studies reported both measures^(30,31)^. Based on the reported UIC or iodine-to-creatinine ratio, there were four studies in severely iodine-deficient children^(27,29,34,35)^, one in severe-to-moderate deficiency^(32)^, one in mild deficiency^(26)^ and three in iodine-sufficient children^(28,30,31)^. None of the studies had a measure of dietary iodine intake (µg/d), and thus the iodine exposure in our review is solely based on measures of urinary iodine excretion (and where available, thyroid volume/goitre rate or Tg as longer term indicators of iodine status).

### Risk of bias and quality assessment

Based on the RoB 2 assessment, the overall risk of bias was low in one of the two RCTs^(33)^ (that in pregnant women); this was judged as having a low risk of bias in all domains (online Supplementary Table S1). The other RCT, in childhood, was judged as having a high risk of bias overall^(32)^; this RCT had some concerns of bias in the randomisation process and the deviations from intended interventions domains, as well as a high risk of bias in the selection of reported results domain. Based on the ROBINS-I assessment, both non-randomised studies of interventions had a serious risk of bias in at least one domain and were thus judged as being at a serious risk of bias overall^(34,35)^ (online Supplementary Table S2). The only cohort study included was of good quality, according to the NOS assessment^(7)^. The majority of cross-sectional studies were of poor quality^(26–28,30)^, one was of fair quality^(29)^ and only one was of good quality^(31)^ (online Supplementary Table S3).

### Maternal iodine exposure in pregnancy and child hearing

Only four studies assessed the association between iodine status or iodine supplementation in pregnancy and child hearing outcomes; only one of these studies was an RCT^(33)^, one was a cohort study^(7)^ and two were case reports^(36,37)^ (Table 1).

**Table 1.** Summary of findings from four studies on the association between maternal iodine status or iodine supplementation during pregnancy and child hearing

Study design				Hearing outcome assessment in children			Child hearing outcomes – results	Overall risk of bias and quality assessment
Study, country	Median UIC (µg/l)	Maternal iodine exposure in study groups (*n*)*	Child iodine status† (time point)	Hearing measure/s	Child age at testing (years)	Assessor		
**RCTs (*n* 1)**								
Gowachirapant, 2017^(33)^, Thailand and India	131 µg/l A: 135 µg/l B: 125 µg/l	A: 200 µg/d iodine from KI tablets (from 10·8 weeks. until delivery) (*n* left/right ear = 115/116) B: placebo tablet (n left/right ear = 117/118)	A: 236 µg/l (at 5·4 years) B: 224 µg/l (at 5·5 years)	Pure-tone audiometry air-conduction (frequency not stated)	5–6 years A: mean 5·4 years B: mean 5·5 years	Blind study-team member	**Pure-tone audiometry:** NS difference overall; left ear (A *v*. B: 15·0 *v*. 13·3 dB, *P* = 0·08) and right ear (A *v*. B: 13·3 *v*. 13·3 dB, *P* = 0·45); 1 child with impaired hearing in the iodine group and 2 children in the placebo group; these were excluded from the analyses	Low risk of bias
**Prospective cohort studies (** ***n*** **1)**								
Hynes, 2017^(7)^, Australia	99 µg/l	A: UIC < 150 µg/l (*n* 30) B: UIC ≥ 150 µg/l (*n* 15)	A: 150 µg/l (at 13–14 years) B: 160 µg/l (at 13–14 years)	**Hearing acuity:** pure-tone audiometry air-conduction; **Binaural processing skills** (ability to use spatial cues for understanding speech in background noise): Listening in Spatialised Noise-Sentences Test (LiSN-S); **Auditory memory:** Number Memory Forwards (NMF) and Number Memory Reversed (NMR) from the Test of Auditory Processing Skills-Third Edition (TAPS-3); **Binaural integration** (the ability to process information presented to both ears simultaneously when the information presented to each ear is different): Dichotic Digits Test (DDT)	13–14 years	N/A	**Hearing acuity (audiometry):** no children had hearing impairment and all audiograms in normal range (i.e. hearing threshold ≤ 20 dB); exact hearing threshold values were not shown; **Binaural processing (LiSN-S): ** neither group reached Speech Reception Thresholds (SRT) for clinical indication of a Central Auditory Processing Disorder (CAPD); NS difference in SRT in unadjusted and all the adjusted models; **Auditory memory (TAPS-3):** NS difference in the NMF test (A *v*. B: 8·7 *v*. 8·3, *P* = 0·708) and in the NMR test (A *v*. B: 8·8 *v*. 9·7, *P* = 0·188); NS difference for both tests in all adjusted models; **Binaural integration (DDT):** compared to ‘sufficient’ group (B), a statistically significantly lower score for ‘deficient’ group (A) for the right ear but not for the left (% correctly repeated digits A *v*. B for left ear: 96·9 *v.* 97·8 %, *P* = 0·29; for right ear: 96·9 *v*. 99 %, *P* = 0·04); association for the right ear persisted in adjusted models; Right ear advantage (REA) (i.e. right ear performed slightly better on DDT) was only observed in the ‘sufficient’ group (B), while the ‘deficient’ group (A) showed similar DDT scores for both ears; the overall REA was NS different between A *v*. B in unadjusted and all adjusted models	Good quality
**Case-reports (*n* 2)**								
Overcash, 2016^(36)^, USA	N/A	12·5 mg/d iodine from iodizyme-HP dietary supplement (5 mg iodine + 7·5 mg iodide) (started in pregnancy until 29 weeks) (*n* 1) **Additional note:** pregnancy was complicated by hypothyroidism: mother took natural TH equiv. to 38 µg T4 from Armour Thyroid (started in pregnancy until 29 weeks.); treated with 75 μg/d Synthyroid (from 29 weeks)	N/A, enlarged fetal neck mass by US (27 weeks.); enlarged fetal thyroid by MRI (29 weeks); smaller thyroid mass by MRI (38 weeks); goitre not palpable (birth); diffusely enlarged thyroid by ultrasound (on DOL 6)	Infant hearing screening and assessment by brainstem auditory evoked response	1st week after birth (DOL 6–8)	N/A, but screening performed in hospital	**Hearing screening and assessment (brainstem auditory evoked response):** infant failed a hearing screening and had bilateral moderate hearing loss for the 500 to 4000 Hz frequency range (likely sensorineural); at 5 weeks the infant was fitted with bilateral hearing aids	Low quality of evidence
Hardley, 2018^(37)^, USA	N/A	12·5 mg/d iodine from supplements (7·5 mg KI + 5 mg free iodine) (12 weeks pre-pregnancy until 21 weeks) (*n* 1) **Additional note: **mother euthyroid pre-pregnancy; mother took 40 mg/d thyroid biotic (12 weeks pre-pregnancy until 21 weeks); treated with 100 µg/d LT4 (from 21 weeks)	N/A, fetal goitre (21 weeks); decreased goitre size (30 weeks); goitre invisible by US (32 weeks); mild thyroid enlargement by MRI (33 weeks); goitre not palpable (birth)	Infant hearing screening	1st week after birth	N/A, but screening performed in hospital	**Hearing screening**: infant had a normal hearing response bilaterally **Comments: **serial intra-amniotic LT4 injections (500 µg/2 weeks) were administered at 22, 24 and 26 weeks	Low quality of evidence

CAPD, Central Auditory Processing Disorder; DDT, Dichotic Digits Test; DOL, day of life; KI, potassium iodide; LiSN-S, Listening in Spatialised Noise-Sentences Test; LT4, levothyroxine; N/A, data not available/reported; NMF, Number Memory Forwards; NMR, Number Memory Reversed; NS, not statistically significant; RCTs, randomised controlled trials; REA, Right Ear Advantage; SRT, Speech Reception Threshold; TAPS-3, Test of Auditory Processing Skills-Third Edition; T4, thyroxine; TH, thyroid hormone; UIC, urinary iodine concentration; US, ultrasound.

* *n* of study groups includes the number of mother–child pairs included in the analysis with the hearing outcomes only; in some studies, the overall sample size in each group is larger (e.g. for other assessed outcomes).

† Child iodine status is reported as median (or mean, if the median was not available; this is indicated by † symbol), UIC (μg/l) and/or urinary iodine-to-creatinine ratio (μg/g) for children with hearing measures only (where available); otherwise, iodine status of a larger sample of children in each study is reported (if available). Where available, iodine status of children of mothers in each study group is reported separately.

In the RCT in mildly iodine-deficient pregnant women (median UIC: 131 µg/l) in India and Thailand, women received either 200 µg iodine/d or placebo from the first trimester until delivery and auditory performance of children (a secondary outcome of the trial) was measured at age 5–6 years^(33)^. There was no statistically significant difference in auditory performance between the iodine and placebo groups^(33)^. Although the median hearing thresholds (measured by audiometry) in the two groups were identical for the right ear (13·3 dB), there was a small difference for the left ear, with a slightly higher hearing threshold in the iodine group (15 dB *v*. 13·3 dB in the placebo group; *P* = 0·08), indicating worse auditory performance.

A cohort study in mildly-to-moderately iodine-deficient pregnant women in Australia (median UIC: 99 µg/l) found that all children had normal hearing (hearing threshold ≤ 20 dB) at 13–14 years regardless of whether their mothers were broadly classified as iodine deficient (UIC < 150 µg/l) or iodine sufficient (UIC ≥ 150 µg/l) during pregnancy^(7)^. This study also found no difference between the two groups of women in the auditory memory and the auditory processing of their children at 13–14 years; neither group of children reached a speech reception threshold indicative of a clinical diagnosis of central auditory processing disorder. There was, however, a statistically significant difference in binaural integration, which reflects a child’s ability to process information presented to both ears simultaneously when each ear is presented with different information, with a lower percentage of correctly repeated digits in children of women with UIC < 150 µg/l *v*. those of women with UIC ≥ 150 µg/l; though this difference was observed for the right ear only (96·9 % *v*. 99 %; *P* = 0·04).

We identified two case reports of pregnant women in the USA who took over fifty times the recommended daily allowance for iodine in pregnancy (USA Institute of Medicine recommended daily allowance: 220 µg/d^(38)^) from dietary supplements^(36,37)^. In both cases, the iodine excess resulted in fetal goitre and hypothyroidism; however, the effect on child hearing was different. In one of the reports, the iodine supplements were discontinued at 29 gestational weeks, but an auditory brainstem response test of the infant indicated sensorineural hearing loss and the infant was fitted with bilateral hearing aids at five weeks^(36)^. By contrast, in the other case report, the iodine supplementation was discontinued around 21 weeks, and the fetus was treated with serial intra-amniotic levothyroxine injections between 22 and 26 weeks; this resulted in fetal euthyroidism and the infant had normal response bilaterally to the neonatal hearing screening^(37)^.

### Iodine exposure in childhood and child hearing

A total of nine studies investigated the association between child iodine exposure and child hearing; one RCT^(32)^, two non-randomised studies of interventions^(34,35)^ and six cross-sectional studies^(26–31)^ (Table 2). The age group of the included children varied from 10 months and 2–4 years^(28)^ to 12–19 years^(31)^; one study had a very wide age range in one of its study groups that also included adults (4–50 years)^(27)^. Overall, eight of the nine studies (89 %) found an association between indicators of iodine status and child hearing, where better iodine status was associated with better hearing^(27–32,34,35)^.

**Table 2.** Summary of findings from nine studies on the association between iodine status or iodine supplementation during childhood and child hearing

Study design			Hearing outcome assessment in children			Child hearing outcomes – results	Overall risk of bias and quality assessment
Study, country, age group	Child iodine status*	Child iodine exposure in study groups (*n*)†	Hearing measure/s	Child age at testing (years)	Assessor		
**RCTs (*n* 1)**							
van den Briel, 2001^(32)^, Benin 7–11 years	20·3 µg/l (before suppl.) 85 µg/l (10 months after suppl.)	A: 540 mg iodine from 1 ml iodised oil orally (single dose) + IS (started 3–4 months after the intervention) (*n* 97) B: placebo – 1 ml poppy seed oil orally (single dose) + IS (started 3–4 months after the intervention) (*n* 100)	Pure-tone audiometry of both ears (frequency: 250, 500, 1000, 2000, 3000, 4000 and 6000 Hz): mean hearing thresholds of both ears at each of the 7 frequencies were shown and hearing results were not given separately per ear	8–12 years‡ (11 months after the intervention and 6–7 months after IS was introduced)	N/A	**Pure-tone audiometry:** IS was introduced while the intervention was ongoing, so hearing results per study group (A *v.* B) were not reported; overall results (A + B) and alternative analysis by groups based on Tg concentration and UIC were reported instead. **Overall results (A + B):** mean hearing threshold = 17·1 dB; 4 % of children had slight hearing impairment (hearing threshold: 26–40 dB; based on the mean value of the better ear at 500, 1000, 2000 and 4000 Hz) and 7 % had slight hearing impairment based on the mean hearing threshold of both ears **Tg as the exposure§:** Tg positively correlated with the mean hearing threshold of the 7 frequencies (*r *= 0·15; *P *= 0·032); children with high Tg (highest tertile) had a higher mean hearing threshold at each of the 7 frequencies than children with low Tg (lowest tertile); differences were statistically significant only at the higher frequencies (≥ 2000 Hz); mean hearing thresholds|| of both ears for low Tg *v.* high Tg: 19·8 *v.* 20·6 dB (NS) at 250 Hz; 21·7 *v.* 22·5 dB (NS) at 500 Hz; 18·2 *v.* 19·9 dB (NS) at 1000 Hz; 12·3 *v.* 15·0 dB (*P* = 0·026) at 2000 Hz; 11·9 *v.* 13·6 dB (*P* = 0·028) at 3000 Hz; 12·1 *v.* 14·1 dB (*P* = 0·052) at 4000 Hz; 15·4 *v.* 17·4 dB (*P* = 0·046) at 6000 Hz **UIC as the exposure: **UIC was not correlated with the mean hearing threshold of the 7 frequencies (*r *= −0·08; *P* = 0·254); children with low UIC (lowest tertile) had higher hearing thresholds at each frequency *v.* children with high UIC (highest tertile) but differences were NS (exact results were not shown)	High risk of bias
**Non-randomised studies of interventions (*n* 2)**							
Azizi, 2005^(34)^, Iran 7–13 years	A: 19 µg/l* B: 102 µg/l* C: 201 µg/l*	A: no iodine suppl. (randomly recruited in 1989, before iodine prophylaxis) (*n* 70) B: 480 mg iodine from 1 ml iodised oil injection (in 1989) (randomly recruited in 1992, 3 years after iodised oil intervention) (*n* 70) C: IS (started in 1993) (randomly recruited in 1999, 7 years after IS exposure) (*n* 72)	Pure-tone audiometry air-conduction (frequency: 500, 1000 and 2000 Hz): mean hearing threshold was calculated as the average of the hearing thresholds at 500, 1000 and 2000 Hz (speech frequencies)	7–13 years A: mean 11 years B: mean 10·8 years C: mean 11·3 years	Technician (same person in all groups); ENT specialist interpreted the results (same person in all groups)	**Pure-tone audiometry: **unclear which ear results relate to, or if results were based on the mean of both ears/the better ear. Overall mean hearing threshold at the speech frequencies was statistically significantly higher in A *v*. B & C (15·8 dB *v*. 10·2 dB & 10·0 dB; *P* < 0·001); NS difference in hearing thresholds in B *v*. C. Proportion with hearing thresholds < 10 dB was significantly lower in A *v*. B & C (0% *v*. 42 & 62%). Proportion with hearing thresholds 10–15 dB was significantly higher in A *v*. C but not *v*. B (A *v*. B *v*. C: 57 *v*. 48 *v*. 28%). Proportion with hearing thresholds > 15 dB was significantly higher in A *v*. B & C (46% *v*. 10 & 10 %; *P* < 0·001).	Serious risk of bias
Wang and Yang, 1985^(35)^, China 7–11 years	A: 19·8 µg/g*(before IS in 1979); 128 µg/g* (1 year after IS); 86 µg/g* (2 years after IS); 125 µg/g* (3 years after IS) B-D: N/A	A: Heba commune (endemic area of ID); IS (started in 1979) (*n* 30) B: Shilong commune (endemic area of ID); IS (started in 1978) (*n* 30) C: Pingliong commune (endemic area of ID); IS (started in 1979) (*n* 30) D: Qianling commune (non-endemic normal control area); IS (started in 1979) (*n* 30)	Pure-tone audiometry air-conduction (frequency: 500, 1000, 2000, 4000 and 8000 Hz): mean hearing threshold was calculated as the average of the hearing thresholds at 500, 1000 and 2000 Hz (speech frequencies)	7–11 years	N/A	**Pure-tone audiometry:** only the results for the right ear were reported. Children in the areas of iodine deficiency A & C had a higher hearing threshold in 1979 (before IS prophylaxis) *v*. children in the control area D; A *v*. C *v*. D: 17·4 *v*. 16·1 *v*. 7·5 dB (hearing threshold before IS prophylaxis was not reported for group B). Children in group A were followed up for 3 years after IS prophylaxis; hearing thresholds decreased significantly 2 and 3 years after IS *v*. before IS: 17·4 dB before IS (in 1979) *v*. 13·9 dB after 1 year (in 1980) *v*. 7·6 dB after 2 years (in 1981) *v*. 8·2 dB after 3 years (in 1982). In group B: hearing threshold decreased significantly from 12·0 dB 1 year after IS (in 1979) to 7·3 dB 3 years after IS (in 1981) (IS started in 1978 in group B – 1 year earlier than other groups). Children in group C (recruited before IS) had a significantly higher hearing threshold than another group of children of the same age and from the same area recruited 3 years after IS: hearing threshold was 16·1 (in group C in 1979) *v*. 8·9 dB (in 30 children of the same age and in the same area in 1982, 3 years after IS).	Serious risk of bias
**Cross-sectional studies (*n* 6)**							
Goslings, 1975^(27)^, Indonesia 5–20 years and 4–50 years	A&B: 16 µg/g* C: 42 µg/g*	A: individuals diagnosed with endemic cretinism (4–50 years) from a community affected by endemic goitre (goitre rate: 85 %) and endemic cretinism (*n* 34) B: individuals without clinical features of endemic cretinism (5–20 years) from the same affected community as group A (*n* 92)¶ C: individuals (5–20 years) from a nearby control area (without endemic goitre) (*n* 54)**	Pure-tone audiometry air-conduction (frequency: 500, 1000, 2000 and 4000 Hz): assessed the number with hearing loss (using hearing threshold cut-offs) and those with normal hearing	A: 5–50 years B: 5–20 years C: 5–20 years	N/A	**Pure-tone audiometry:** hearing thresholds not shown at all frequencies tested and for each ear for the study groups; bilateral hearing loss at 4000 Hz reported for each group. % with normal hearing (< 20 dB) in A *v*. B *v*. C: 8·8 *v*. 97·8 *v*. 98·1 %; % with bilateral hearing loss at 4000 Hz (≥ 20 dB) in A *v*. B *v*. C: 91·2 (20–60 + dB) *v*. 2·2 (20–30 dB) *v*. 1·9 % (20–30 dB) (NS difference between B *v*. C); hearing loss in most subjects in group A was more severe at higher frequencies than lower frequencies (hearing thresholds at each frequency not shown for all individuals in group A); there were 5 deaf mute subjects in group A *v*. none in groups B and C	Poor quality
Todd, 1988^(26)^, Zimbabwe 9–16 years	A: 83 µg/g†† B: 88 µg/g††	A: goitre (*n* 59) B: no goitre (*n* 62)	Pure-tone audiometry air-conduction of both ears (frequency: 125, 250, 500, 1000, 4000 and 8000 Hz)	9–16 y	Experienced audiometrist	**Pure-tone audiometry: **results not reported per study group; all children tested heard all frequencies at 0 dB in both ears and nearly all (*n* 111) children heard all frequencies at −10 dB; all children had normal hearing	Poor quality
Valeix, 1994^(28)^, France 10 months and 2–4 years	181 µg/l (10-month-olds) 134 µg/l (2-year-olds) 116 µg/l (4-year-olds)	A: UIC < 100 µg/l (n = N/A)‡‡ B: UIC ≥ 100 µg/l (n = N/A)‡‡	Pure-tone audiometry of both ears (frequency: 250, 500, 1000, 2000 and 4000 Hz); conditioned orientation reflex used (binaural testing) if child < 4 years: mean hearing threshold calculated as average of the hearing thresholds at 500, 1000 and 2000 Hz (speech frequencies)	10 months 2–4 years	Audiologist	**Pure-tone audiometry:** hearing thresholds not reported per UIC group; overall results (A + B) were presented as a correlation between mean hearing thresholds of both ears (at 4000 Hz and at the speech frequencies) and UIC for each age group. **Overall results (A + B):** in 4-year-olds, hearing threshold at 4000 Hz correlated with UIC (*r *= 0·10, *P* < 0·02) and the correlation between UIC and the average hearing threshold at the speech frequencies (500, 1000 and 2000 Hz) was weak and NS (*r *= 0·03, *P* < 0·25); overall, hearing loss was more severe in children with UIC < 100 µg/l *v*. children with UIC > 100 µg/l (exact results not shown per UIC group); similar results were seen for the 2-year-olds (exact results were not shown); results for the 10-month-old children not reported	Poor quality
Azizi, 1995^(29)^, Iran 6–16 years	A: 19·8 µg/g §§,* B: 18 µg/g* C: 66 µg/g*	A: Kiga village (endemic goitre area; 5 and 95 % with goitre grades 1 and 2) (*n* 95) B: Keshar village (endemic goitre area; 33 and 64 % with goitre grades 1 and 2) (*n* 103) C: Tehran (46 and 22 % with goitre grades 1 and 2) (*n* 73)	Pure-tone audiometry air-conduction (frequency: 500, 1000 and 2000 Hz): mean hearing threshold was calculated as the average of the hearing thresholds at 500, 1000 and 2000 Hz (speech frequencies)	6–16 years	ENT specialist	**Pure-tone audiometry:** unclear which ear results relate to, or if based on mean of both ears/better ear. Mean hearing threshold significantly higher in A *v*. B & C: 15·4 *v*. 13·2 (*P* < 0·005) & 12·4 dB (*P* < 0·001); % with abnormal hearing in A *v*. B *v*. C: 44 *v*. 15 *v*. 2 %; in group A: 47 had high tone loss, 26 had conduction problems and 5 had sensorineural deficit (this breakdown was not reported for groups B&C)	Fair quality
Soriguer, 2000^(30)^, Spain 6–16 years	120 µg/l 117 µg/g	A1: palpable goitre+ UIC < 50 µg/l (*n* 15) A2: palpable goitre+ UIC 51–100 µg/l (*n* 20) A3: palpable goitre+ UIC 101–150 µg/l (*n* 18) A4: palpable goitre+ UIC > 150 µg/l (*n* 18) B1: no palpable goitre + UIC < 50 µg/l (*n* 13) B2: no palpable goitre + UIC 51–100 µg/l (*n* 16) B3: no palpable goitre + UIC 101–150 µg/l (*n* 13) B4: no palpable goitre + UIC > 150 µg/l (*n* 10)	**Pure-tone audiometry air-conduction** of both ears (frequency: 125, 250, 500, 1000, 2000, 4000 and 8000 Hz) **Bone-conduction hearing thresholds** of both ears (frequency: 250, 500, 1000, 2000 and 4000 Hz): performed only in those who had air-conduction hearing threshold > 20 dB at any frequency	6–16 years mean: 10·6 years	N/A	**Pure-tone audiometry air conduction||||: **only results for right ear were reported; authors state that the results for left ear were similar. Children with goitre (A1–A4): significant inverse relationship between UIC and auditory thresholds at all frequencies (except 8000 Hz); hearing threshold in A1 *v*. A2 *v*. A3 *v*. A4: 24·4 *v*. 19·0 *v*. 17·6 *v*. 16·8 dB (*P* = 0·05) at 125 Hz, 27·3 *v*. 21·1 *v*. 19·1 *v*. 18·3 dB (*P* = 0·01) at 250 Hz, 30·0 *v*. 21·2 *v*. 19·7 *v*. 18·5 dB (*P* = 0·01) at 500 Hz, 24·4 *v*. 15·5 *v*. 12·9 *v*. 13·7 dB (*P* = 0·01) at 1000 Hz, 17·2 *v*. 9·1 *v*. 9·3 *v*. 10·3 dB (*P* = 0·05) at 2000 Hz, 20·4 *v*. 9·1 *v*. 10·9 *v*. 10·0 dB (*P* = 0·05) at 4000 Hz, 16·4 *v*. 11·5 *v*. 11·0 *v*. 12·7 dB (NS) at 8000 Hz Children without goitre (B1–B4), hearing threshold was higher in the groups with lower UIC but the differences were smaller, and the association was NS at any frequency; hearing threshold in B1 *v*. B2 *v*. B3 *v*. B4: 19·7 *v*. 18·7 *v*. 17·9 *v*. 18·9 dB (NS) at 125 Hz, 22·5 *v*. 22·5 *v*. 20·7 *v*. 21·5 dB (NS) at 250 Hz, 23·0 *v*. 23·4 *v*. 18·9 *v*. 20·4 dB (NS) at 500 Hz, 19·2 *v*. 16·7 *v*. 15·6 *v*. 18·1 dB (NS) at 1000 Hz, 14·3 *v*. 11·3 *v*. 12·1 *v*. 12·3 dB (NS) at 2000 Hz, 14·3 *v*. 9·1 *v*. 11·1 *v*. 8·5 dB (NS) at 4000 Hz, 15·3 *v*. 12·1 *v*. 11·9 *v*. 13·1 dB (NS) at 8000 Hz In multiple regression, UIC was negatively associated with air conduction hearing threshold at 2000 Hz only in children with UIC ≤ 100 µg/l (*P* = 0·03); in children with goitre + UIC ≤ 100 µg/l: Tg, UIC and age accounted for 75 % of variance in air-conduction hearing threshold at 2000 Hz. **Bone conduction||||:** analysis shown for children with UIC ≤ 100 µg/l (A1–A2 & B1–B2) *v.* > 100 µg/l (A3–A4 & B3–B4); only a small number had bone-conduction data (UIC ≤ 100 µg/l: n 8; UIC > 100 µg/l: n 7). In children with goitre (A1–A4), hearing threshold was higher in those with UIC ≤ 100 µg/l *v*. > 100 µg/l at 500 and 1000 Hz for the right ear and at 500 and 4000 Hz for the left ear; hearing threshold in UIC ≤ 100 µg/l *v*. > 100 µg/l for right/left ear: 15·1–16·0 *v*. 8·4–11·1 (NS)/15·1 *v*. 5·0 dB (NS) at 250 Hz, 16·0 *v*. 10·9 (*P *≤ 0·05)/15·1–16·0 *v*. 8·4–11·1 dB (*P* ≤ 0·05) at 500 Hz, 15·1–16·0 *v*. 8·4–11·1 (*P* ≤ 0·05)/15·1–16·0 *v*. 8·4–11·1dB (NS) 1000 Hz, 10·8 *v*. 10·0 (NS)/15·1–16·0 *v*. 8·4–11·1 dB (NS) at 2000 Hz, 18·8 *v*. 12·2 (NS)/15·1–16·0 *v*. 8·4–11·1 dB (*P* ≤ 0·05) at 4000 Hz. In children without goitre (B1–B4): NS association between hearing threshold and UIC at any frequency; hearing threshold in UIC ≤ 100 µg/l *v*. > 100 µg/l for right/left ear: 8·4 *v*. 5·0 (NS)/8·0 *v*. 15·0 dB (NS) at 250 Hz, 8·7 *v*. 7·5 (NS)/10·0 *v*. 16·0 dB (NS) at 500 Hz, 13·0 *v*. 8·3 (NS)/8·5 *v*. 8·8 dB (NS) at 1000 Hz, 13·5 *v*. 12·5 (NS)/12·7 *v*. 13·3 dB (NS) at 2000 Hz, 12·8 *v*. 11·4 (NS)/9·4 *v*. 12·8 dB (NS) at 4000 Hz. **Tg as the exposure||||, ¶¶: **hearing thresholds by Tg group (Tg ≤ 10 *v.* > 10 ng/ml) were reported for the total sample (A1–B4). (1) Air conduction: children with Tg > 10 ng/ml had higher auditory thresholds at almost all frequencies *v*. those with Tg ≤ 10 ng/ml (at 125 Hz in both ears; at 250 Hz in left ear; at 500 Hz in both ears; at 1000 Hz in left ear; at 2000 Hz in left ear; at 4000 Hz in both ears; at 8000 Hz in left ear); Tg ≤ 10 *v*. Tg > 10 ng/ml: (right ear) 20·3 *v*. 24·2 dB (NS) at 250 Hz; 21·1 *v*. 26·0 dB (*P* = 0·05) at 500 Hz; 11·5 *v*. 13·9 dB (NS) at 2000 Hz; 10·0 *v*. 15·2 dB (*P* = 0·05) at 4000 Hz; (left ear) 15·0 *v*. 19·0 dB (*P* = 0·03) at 1000 Hz; 10·0 *v*. 13·4 dB (*P* = 0·03) at 4000 Hz; overall range for both ears: 10–21 dB for Tg ≤ 10 ng/ml *v*. 13–26 dB for Tg > 10 ng/ml. (2) Bone conduction: auditory thresholds at 1000 and 4000 Hz for both ears were lower when Tg ≤ 10 *v*. Tg > 10 ng/ml: (right ear) 8·6 *v*. 16·0 dB (*P* ≤ 0·05) at 1000 Hz; 10·6 *v*. 18·0 dB (*P* ≤ 0·05) at 4000 Hz; (left ear) 7·5 *v*. 10·5 dB (NS) at 250 Hz; 12·2 *v*. 13·2 dB (NS) at 500 Hz; 12·5 *v*. 14·0 dB (NS) at 2000 Hz; 9·5 *v*. 12·3 dB (*P *≤ 0·05) at 4000 Hz; overall range for both ears: 7·5–13·7 dB for Tg ≤ 10 ng/ml *v*. 10·5–18 dB for Tg > 10 ng/ml (3) Multiple regression: Tg positively associated with air- and bone conduction hearing thresholds only in children with goitre (*P* < 0·001; *P* = 0·01) and with air conduction threshold only in those with UIC ≤ 100 µg/l (*P* < 0·001) Thyroid volume (TV) as the exposure***: in adjusted logistic regression, children with TV > 95th percentile had higher odds of hearing threshold > 20 dB (OR (95 % CI): 3·86 (2·59, 5·10)); higher odds of hearing threshold > 20 dB with goitre, defined by palpation, (OR (95 % CI): 1·97 (1·34, 2·52)) or when TV > 75th percentile (OR (95 % CI): 3·06 (2·19, 3·92))	Poor quality
Scinicariello and Buser, 2018^(31)^, USA 12–19 years	154 µg/l* 130 µg/g*,†††	A: UIC < 100 µg/l (*n* 353) B: UIC 100–199 µg/l (*n* 396) (Ref.) C: UIC ≥ 200 µg/l (*n* 503)	Pure-tone audiometry air conduction (frequency: 500, 1000, 2000, 4000 Hz (speech frequencies for SFHL) and 3000, 4000, 6000 Hz (high frequencies for HFHL)): hearing loss (SFHL/HFHL) was defined as the average of all speech/high frequencies in the better ear > 15 dB	12–19 years mean: 15·3 years	N/A	**Pure-tone audiometry:** an increased risk of speech-frequency hearing loss (SFHL) in A *v*. B but not in C *v*. B in adjusted models – Model 2, OR (95 % CI): A *v*. B 2·10 (1·04, 4·26); C *v*. B 1·23 (0·52, 2·93) NS; NS association of UIC with high-frequency hearing loss (HFHL) – Model 2, OR (95 % CI): A *v*. B 1·09 (0·61, 1·97) NS; C *v*. B 1·03 (0·61, 1·74) NS Group A split: < 50 (*n* 111) and 50–99 µg/l (*n* 242); increased risk of SFHL in children and adolescents with UIC < 50 µg/l *v*. B but not in those with UIC 50–99 µg/l *v*. B – Model 2, OR (95 % CI): UIC < 50 *v*. B 5·52 (1·94, 15·68); UIC 50–99 µg/l *v*. B 1·60 (0·71, 3·62) NS; having UIC < 50 or 50–99 µg/l was not associated with HFHL – Model 2, OR (95 % CI): UIC < 50 *v*. B 2·14 (0·91, 5·05) NS; UIC 50–99 µg/l *v*. B 0·90 (0·46, 1·17) NS	Good quality

dB, decibels; ENT, ear, nose and throat; HFHL, high-frequency hearing loss; ID, iodine deficiency; IS, iodised salt; N/A, data not available/reported; NS, not statistically significant; RCTs, randomised controlled trials; Ref., reference group; SFHL, speech-frequency hearing loss; Tg, thyroglobulin; TV, thyroid volume; UIC, urinary iodine concentration.

* Child iodine status is reported as median (or mean, if the median was not available; this is indicated by * symbol) UIC (μg/l) and/or urinary iodine-to-creatinine ratio (μg/g) for children with hearing measures only (where available); otherwise, iodine status of a larger sample of children in each study is reported (if available). Where available, iodine status of children in each study group is reported separately.

† *n* of study groups includes the number of children included in the analysis with the hearing outcomes only; in some studies, the overall sample size in each group is larger (e.g. for other assessed outcomes).

‡ Child age at hearing assessment was estimated in the study by van den Briel^(32)^ as only child age at the beginning of the study was reported; children were 7–11 years at the beginning of the study, and the hearing outcome was measured one year later, resulting in an estimated child age at testing of 8–12 years.

§ Median Tg concentration was 215 pmol/l before supplementation (at baseline) and 95 pmol/l 10 months after supplementation (6–7 months after iodised salt was introduced in the population) in the study by van den Briel^(32)^.

|| Mean hearing thresholds were estimated from a bar chart in the study by van den Briel^(32)^.

¶ One subject from group B in the study by Goslings^(27)^ was excluded due to eardrum perforations (total n for analysis in group B = 91).

** Two subjects from group C in the study by Goslings^(27)^ were excluded due to eardrum perforations (total n for analysis in group C = 52).

†† Not all children who underwent hearing testing (*n* 121) had iodine-to-creatinine ratio measured in the Todd study^(26)^; the iodine-to-creatinine ratio reported was measured in a total of 61 children (n=31 in group A and n=30 in group B), of these 43 had hearing tested.

‡‡ Sample size per study group was not reported in the study by Valeix^(28)^; total sample size for each age group of children recruited was 456 (10 months), 368 (2 years) and 398 (4 years).

§§ Iodine-to-creatinine ratio in group A was measured in a larger sample of children (*n* 190) than the sample with data from the hearing testing (*n* 95) in the study by Azizi^(29)^.

|||| Actual values for the hearing thresholds were not reported in the study by Soriguer^(30)^, values shown were estimated (where possible) from the line graphs in the paper; in the cases where it was difficult to distinguish between the lines, ranges are provided (undistinguishable values for some frequencies were not reported).

¶¶ Median Tg concentration in the total sample was 9·0 ng/ml in the study by Soriguer^(30)^.

*** Mean thyroid volume was 7·0 ml in children with palpable goitre (A1–A4) and 4·6 ml in children with no palpable goitre (B1–B4) in the study by Soriguer^(30)^.

††† Iodine-to-creatinine ratio was not reported in the study by Scinicariello and Buser^(31)^,but we calculated this using the reported mean value for urinary creatinine (118 mg/d).

The only RCT in children was conducted in Benin, West Africa; it administered a single dose of iodised oil or a placebo to children of 7–11 years who were moderately-to-severely iodine deficient (median UIC: 20 µg/l) and measured their hearing thresholds by pure-tone audiometry eleven months later^(32)^. Despite being set up as an RCT, this study did not compare hearing thresholds between the study groups because the whole population began to have access to iodised salt three to four months after the start of the intervention (i.e. both groups had been exposed to iodised salt for 6–7 months at the time of the hearing test). Iodine status of the total sample improved from baseline, and children were mildly deficient at the end of the study (median UIC: 85 µg/l). The authors of this study performed an alternative analysis of the data from the total sample; this showed that thyroglobulin (Tg) (a marker positively correlated with the severity of iodine deficiency^(39)^) was positively correlated with the mean hearing threshold of the seven frequencies tested (between 250 and 6000 Hz); although statistically significant, this correlation was only weak (*r* = 0·15; *P* = 0·032)^(32)^. Children with Tg in the highest tertile had a higher mean hearing threshold at each of the seven frequencies than children with Tg in the lowest tertile, but these differences were statistically significant only at the higher frequencies (≥ 2000 Hz) (Table 2). By contrast, UIC was not correlated with the mean hearing threshold and although children with lower UIC had a higher mean hearing threshold at each frequency, the differences did not reach statistical significance. Children with a higher hearing threshold (i.e. those who had worse hearing) also performed worse on the battery of mental development tests^(32)^.

Both studies of iodine interventions in children in Iran^(34)^ and China^(35)^ showed that the administration of iodised oil or iodised salt resulted in a significant reduction in the hearing thresholds (i.e. better hearing). The study in Iranian school-age children (7–13 years) showed that the mean hearing threshold at the speech frequencies (500, 1000 and 2000 Hz) was statistically significantly lower in children recruited three years after the administration of iodised oil (in 1992, mean UIC: 102 µg/l; 10·2 dB) and seven years after the introduction of iodised salt in Iran (in 1999, mean UIC: 201 µg/l; 10·0 dB) than in the children recruited prior to any iodine prophylaxis (in 1989, mean UIC: 19 µg/l; 15·8 dB)^(34)^. Moreover, a significantly lower proportion of children with hearing thresholds > 15 dB was observed in 1992 (three years after supplementation of all children with iodised oil injections) and in 1999 (seven years after iodised-salt exposure) than in 1989 (prior to iodine prophylaxis); 10 % in both 1992 and 1999 *v*. 46 % in 1989. The study in China found that the mean hearing threshold at the speech frequencies of school children (7–11 years) who lived in two endemic areas of severe iodine deficiency was higher than that of children in a non-endemic control area (17·4 and 16·1 dB *v*. 7·5 dB, respectively)^(35)^. Children in one of the iodine-deficient areas were followed up for three years after the introduction of iodised salt; the mean hearing threshold of these children decreased significantly two and three years after introducing iodised salt (7·6 and 8·2 dB, respectively, *v*. 17·4 dB before iodine prophylaxis) to a value similar to that of children living in the control non-endemic area^(35)^. The improvement in hearing thresholds after supplementation with iodised salt was also accompanied by a reduction in goitre rate (before *v*. three years after iodised salt: 32 *v*. 6 %), an increase in the mean iodine-to-creatinine ratio (before *v*. three years after iodised salt: 20 *v*. 125 µg/g) and a normalisation of thyroid function tests. To account for the effect of advancing age in this study, in one of the endemic areas, two groups of children were recruited – one before iodine prophylaxis and one three years after the introduction of iodised salt but of the same age. Children recruited before iodised-salt supplementation had a significantly higher hearing threshold than the group of children of the same age and from the same area recruited three years after supplementation (hearing threshold 16·1 *v*. 8·9 dB, respectively)^(35)^.

Only one of the six cross-sectional studies did not find any association between measures of child iodine status and hearing; that study examined 9–16-year-old children (*n* 121) either with or without goitre and found that all children had normal hearing as measured by pure-tone audiometry^(26)^. Although the study groups differed based on the presence of goitre (a historical long-term marker of iodine intake), the median iodine-to-creatinine ratio was similar (83 *v*. 88 µg/g).

Of the five cross-sectional studies that found an association, three found an association between UIC and hearing thresholds in children or adolescents^(28,30,31)^, one study reported an association of Tg and thyroid volume with hearing thresholds^(30)^ and two studies found an association between iodine-to-creatinine ratio and the presence of endemic goitre in the area of residence and child hearing thresholds^(27,29)^. All these studies suggested that lower iodine status (indicated by the exposures above) was associated with worse hearing in children (indicated by higher hearing thresholds).

A study in France found a correlation between UIC and hearing threshold in 4-year-old children (median UIC: 116 µg/l), where higher UIC indicated better hearing; however, it was weak and was statistically significant only when the hearing threshold was measured at 4000 Hz (*r* = 0·10; *P* < 0·02) and not at the speech frequencies (500, 1000 and 2000 Hz)^(28)^. Overall, higher hearing threshold was observed in the children with UIC < 100 µg/l than in those with UIC > 100 µg/l^(28)^, but the exact results for each of these groups were not reported.

A cross-sectional study in Spanish school-age children (6–16 years) reported a statistically significant negative association between spot-UIC and air-conduction auditory thresholds at all tested frequencies (except at 8000 Hz) in the children with palpable goitre^(30)^. In this group, the hearing thresholds (at 125–4000 Hz) were in the range of 17·2–30·0 dB in children with UIC < 50 µg/l and between 9·3–19·7 dB in children with UIC 101–150 µg/l. In children without palpable goitre, a higher hearing threshold was seen in children with lower UIC but the differences were smaller and not statistically significant. The results for the bone-conduction auditory thresholds were mixed in children with goitre, whereas, similarly to the air-conduction results, there was no statistically significant association with UIC in children without goitre. Children with Tg > 10 ng/ml had higher auditory thresholds for both ears at almost all frequencies than those with Tg < 10 ng/ml; results were similar for both air-conduction (overall hearing threshold range for both ears: 10–21 dB for Tg ≤ 10 ng/ml *v*. 13–26 dB for Tg > 10 ng/ml) and bone-conduction testing (overall hearing threshold range for both ears: 7·5–13·7 dB for Tg ≤ 10 ng/ml *v*. 10·5–18 dB for Tg > 10 ng/ml). In multiple regression analysis, Tg was positively associated with hearing thresholds only in children with goitre and only in those with UIC ≤ 100 µg/l. UIC was negatively associated with air-conduction hearing threshold at 2000 Hz only in children with UIC ≤ 100 µg/l. In children with goitre and UIC ≤ 100 µg/l, Tg, UIC and age accounted for 75 % of the variance in the air-conduction hearing threshold at 2000 Hz. In adjusted model, children with thyroid volume > 95th percentile were nearly four times (OR: 3·86) more likely to have a hearing threshold > 20 dB. Higher odds of hearing threshold > 20 dB were also seen when goitre was defined by palpation (OR: 1·97) or when thyroid volume > 75th percentile (OR: 3·06)^(30)^.

A study of USA adolescents (12–19 years) found that in adjusted analyses, those with UIC < 100 µg/l had an increased risk of speech-frequency hearing impairment (defined as the average hearing threshold of all speech frequencies in the better ear > 15 dB) compared with those with UIC 100–199 µg/l (OR: 2·10)^(31)^. The association was even more pronounced in those with UIC < 50 µg/l compared with the same reference group (i.e. UIC 100–199 µg/l) (OR: 5·52). In this study, UIC was not associated with high-frequency hearing impairment.

Two of the six cross-sectional studies investigated hearing thresholds in areas of severe iodine deficiency and endemic goitre in comparison with non-endemic/control areas^(27,29)^. A study in Indonesia included both children and adults (4–50 years) in the following groups: those from a community affected by endemic goitre (mean iodine-to-creatinine ratio: 16 µg/g) with cretinism (Group A) and without cretinism (Group B), as well as individuals from a nearby non-endemic goitre control area (mean iodine-to-creatinine ratio: 42 µg/g) (Group C). We included this study because although it contained data on iodine exposure in adults, most participants in groups B and C were children (age range: 5–20 years). Group A included participants up to 50 years, but these individuals had cretinism, which would have likely indicated exposure to iodine deficiency during pregnancy. They found that the proportion with bilateral hearing impairment (defined as a hearing threshold measured by pure-tone audiometry ≥ 20 dB) was significantly higher in Group A than in Group B or C (91·2 % *v*. 2·2 % *v*. 1·9 %, respectively). There was no statistically significant difference between Groups B and C^(27)^. By contrast, a study in Iranian school-age children (6–16 years) without visible signs of endemic cretinism (i.e. similar to Group B in the previous study^(27)^) found a higher proportion of children with abnormal hearing function in two endemic-goitre areas with mean iodine-to-creatinine ratio 18–20 µg/g (Groups A and B) than in another area with mean iodine-to-creatinine ratio 66 µg/g (Group C) (44 and 15 % *v*. 2 %, respectively)^(29)^. The mean hearing threshold at the speech frequencies (500, 1000 and 2000 Hz) was also statistically significantly higher in the severely iodine-deficient children (Group A) than in those who were mildly-moderately deficient (Group C) (15·4 *v*. 12·4 dB, respectively). Notably, over 90 % of children in Group A also had a visible goitre.

## Discussion

This systematic review provides an overview of the available evidence on the association between iodine exposure during development (*in utero* and/or in childhood) and hearing outcomes in children. Overall, limited and poor-quality data were available, thus affecting our ability to draw firm conclusions. The fact that there is a lack of good-quality evidence is an important finding that highlights the need for further research in this area. We therefore discuss the current knowledge gaps and provide directions for future research.

### Iodine status during pregnancy and child hearing

Studies investigating associations between maternal iodine status during pregnancy and child hearing are lacking; only four such studies were identified in this review and only one of these was an RCT^(33)^. That RCT conducted in India and Thailand found no association between iodine supplementation in mildly iodine-deficient pregnant women and offspring hearing at five years^(33)^. Notably, auditory performance was not the primary outcome in this trial and the target sample size of mother–child pairs was estimated to detect differences between study groups in the primary outcome (i.e. IQ scores). Although iodine supplementation significantly increased maternal UIC, it did not result in major differences in thyroid function tests between the iodine and placebo groups. The iodine status of the placebo group also improved during the study (second and third trimester median UIC were above the WHO cut-off of 150 µg/l^(40)^ indicating iodine sufficiency in pregnancy); this might have contributed to the null findings. Furthermore, the setting of this RCT limits interpretation of the results as the women in India were iodine sufficient at baseline and therefore improvements in auditory performance would not be expected with the intervention in this iodine-sufficient group^(41)^. Although the authors have subsequently re-analysed just the data of the iodine-deficient pregnant women from Thailand, this did not include analysis of the auditory outcomes^(42)^.

The only cohort study in pregnant women found mostly no associations between maternal iodine status (UIC) and child hearing outcomes at 13–14 years, though children of iodine-deficient women had lower scores for binaural integration (processing information presented to both ears simultaneously)^(7)^. This study was in a setting of mild-to-moderate iodine deficiency and was of good quality; however, it only included 45 mother–child pairs. Evidence in settings of mild-to-moderate iodine deficiency in pregnancy is therefore lacking.

Excessive iodine intake in the diet can also have negative consequences for the fetus and neonate, who might be at risk of iodine-induced thyroid dysfunction^(43)^. In the two case reports included in this review^(36,37)^, both pregnant women were taking an extremely high dose of iodine from dietary supplements (over 50 times the recommended daily allowance of 220 µg/d^(38)^); however, this resulted in hearing problems in the newborn only in one of these cases^(36)^. The timing of discontinuation of the supplement (at 21^(37)^ *v*. 29 weeks^(36)^) and the administration of intra-amniotic levothyroxine injections in one of the cases^(37)^ might account for the different outcomes. In one of the reports (where the newborn had hearing problems)^(36)^, the mother was also hypothyroid during pregnancy, while the mother in the other case report was euthyroid^(37)^. More evidence on the safe dose of iodine supplements in pregnancy is needed.

The development of the auditory system is prolonged and continues after pregnancy into childhood^(15)^. It is unclear which developmental periods are most sensitive to the effects of iodine deficiency and whether child hearing impairment resulting from iodine deficiency in pregnancy could be reversed by the correction of iodine deficiency in childhood. Some studies included in this review showed an improvement in hearing thresholds after iodine supplementation in childhood^(34,35)^, suggesting that hearing impairment might be reversible in later childhood. The RCT^(33)^ and cohort study^(7)^ in pregnant women who were mildly-to-moderately iodine deficient did not find an association between maternal iodine exposure and child hearing threshold, but notably the children in these studies were iodine sufficient. By contrast, it has been suggested that the processes of auditory development most susceptible to changes in T_3_ availability occur mostly during pregnancy and to a lesser extent postnatally^(12)^. The limited existing evidence in pregnancy, however, does not provide information about the critical timing of maternal iodine deficiency in relation to auditory development.

Studies in pregnant women with child hearing outcomes are needed to expand the body of evidence in this area. Ideally, these would be RCTs, but this study type is becoming increasingly challenging to conduct as more countries introduce iodine-supplementation recommendations for pregnant women and therefore there are ethical concerns over having a placebo group. In the absence of further RCTs, cohort studies could provide more data on the association between iodine status in pregnancy and offspring hearing function. We identified only one such study where iodine status was assessed throughout pregnancy and results were not reported according to trimester^(7)^; it is possible that the effects of iodine depend on the period of exposure during auditory development. This concept could be explored in future cohort studies with measures of iodine status at different time points in pregnancy.

### Iodine status in childhood and hearing

Most studies in this review were focussed on iodine exposure during childhood; the majority (five of nine) were conducted in areas of endemic goitre and severe or severe-to-moderate iodine deficiency^(27,29,32,34,35)^, just one study was in a population with mild deficiency^(26)^ and three were in areas of iodine sufficiency^(28,30,31)^. The negative effects of severe iodine deficiency, including mental retardation, goitre and deafness, are well documented^(2,3)^, whereas the consequences of exposure to milder forms of deficiency are less clear. Nowadays, severe iodine deficiency is rare, whereas mild-to-moderate deficiency is more prevalent^(44)^; this highlights the need for more studies on hearing in mild-to-moderate deficiency.

The studies in children were predominantly cross-sectional and five of six found that lower iodine status indicated by various measures was associated with higher hearing thresholds in children (i.e. worse hearing)^(27–31)^. In three of the studies, the associations were based on UIC^(28,30,31)^, though in one of the studies, the associations were weak and not present at all hearing frequencies^(28)^, and in another study – only seen in children with goitre^(30)^. As these studies were cross-sectional, it is important to note that the low iodine status in childhood might also be a marker of inadequate iodine exposure *in utero*; the hearing impairment observed in children might be as a result of suboptimal iodine status of their mothers who also resided in these severely iodine-deficient areas during pregnancy.

Two cross-sectional studies (Indonesia and Iran) compared hearing thresholds of individuals in areas of severe iodine deficiency and endemic goitre with those in control areas^(27,29)^. The study in Indonesia found that the proportion with bilateral hearing impairment was significantly higher in children with cretinism in the endemic goitre area, compared with children either without cretinism from the same area, or from the control area, while there was no difference between children without cretinism and the control area^(27)^. This might suggest that hearing impairment is only likely when children have cretinism; however, children in the control area were still moderately iodine-deficient and their iodine status was not much higher than that of children in the endemic-goitre area^(27)^. By contrast, the study in Iran found a higher mean hearing threshold and a higher proportion with abnormal hearing in children without cretinism from endemic-goitre areas *v*. in children from a control area^(29)^; notably, the difference in iodine status between the goitre and control areas was greater than between the groups of children in Indonesia^(27)^. The study in Iran suggests that severe iodine deficiency in children might affect hearing thresholds, even without the presence of clinical cretinism.

The majority of these cross-sectional studies were of poor quality and most did not adjust for confounders, so it is unclear whether the observed associations are independent of socio-demographic characteristics (e.g. age) and other potential confounders (e.g. intake of other nutrients that are important for both auditory function and thyroid metabolism, such as iron^(18,45)^). Moreover, different levels of otitis media between study groups could explain some of the differences in hearing in observational studies^(18)^. Better-quality studies in children are needed to strengthen the evidence observed in previous studies.

The RCT in children did not report the difference in hearing thresholds between the iodine (as iodised oil) and placebo groups because iodised salt was introduced in the population during the study^(28)^. However, it did report that Tg was positively associated with hearing thresholds; children with Tg in the highest tertile (i.e. indicative of low iodine status) had higher hearing thresholds (i.e. worse hearing) than those in the lowest tertile, though differences were small (around 1–2 dB). By contrast, UIC was not associated with hearing thresholds, which could be because Tg is a longer-term marker of iodine intake (preceding weeks to months) than UIC, which reflects recent iodine intake (last 24–48 h)^(39)^. A cross-sectional study also found that children with Tg > 10 ng/ml had higher hearing thresholds than children with Tg ≤ 10 ng/ml (around 2–5 dB different at most frequencies), whereas UIC was negatively associated with hearing thresholds only in children with goitre^(30)^.

### The implications of impaired hearing in relation to iodine

The hearing results in the included studies should be considered in the context of the WHO grades of hearing impairment, hearing thresholds of 20 dB or lower in both ears is defined as ‘normal’ hearing^(18)^. In some studies, most hearing thresholds were below 20 dB^(30,32)^. Both intervention studies in severely iodine-deficient children showed that the administration of iodised oil^(34)^ or iodised salt^(35)^ resulted in a significant reduction in the hearing thresholds. However, the mean hearing threshold at the speech frequencies (i.e. 500, 1000 and 2000 Hz) was below 20 dB (i.e. 15·8 dB^(34)^ and 17·4 dB^(35)^) even in the non-supplemented groups. In a cross-sectional study, children from an endemic-goitre area had a mean hearing threshold at the speech frequencies of 15·4 dB^(29)^, and in another, most hearing thresholds were above 20 dB only in children with goitre and with UIC < 50 µg/l^(30)^. Taken together, these results suggest that in individuals without clinical features of endemic cretinism, the effect of iodine deficiency on hearing might be relatively subtle; however, this does not mean that the effects are unimportant. It is possible that even small differences in hearing thresholds or mild hearing impairment may have consequences for cognitive outcomes^(46)^. This is supported by data from a study in children with average hearing thresholds within the normal range that showed that poorer hearing threshold was associated with lower IQ (7–13 years), poorer scores for reading (11–15 years) and language comprehension and expression (3–9 years)^(47)^.

Previous studies have shown a link between maternal mild-to-moderate iodine deficiency and offspring reading^(4)^, IQ scores^(4)^, language skills^(5)^ and school performance^(6,7)^. As previously noted by Hay *et al.* ^(48)^, mild-to-moderate maternal iodine deficiency tends to be associated with difficulties in processing information quickly, and these effects are usually captured by measures of verbal processing and language. However, the role of child hearing in these reported associations is unclear; for instance, whether these are separate negative effects of iodine deficiency, or whether the association between maternal iodine status and child cognition reported in previous studies might be partly explained by suboptimal child hearing ability. Alternatively, poorer cognitive ability could explain poorer performance on audiometry, a subjective, psychophysical test which requires a child to sustain attention and process sound to measure their hearing threshold accurately.

Hearing problems in children can have an effect on cognitive, academic and social skills (e.g. communication and spoken language)^(18)^. For instance, a study in the UK ALSPAC cohort showed that a significantly lower proportion of children with hearing impairment achieved the top grades of A*–C on five or more General Certificate of Secondary Education exams at 16 years (including English and Maths) compared with children with normal hearing and vision (64 *v*. 73 %)^(49)^, though the association was attenuated after adjustment for IQ. The relationship between IQ and hearing has been documented previously, even slight-to-moderate sensorineural hearing loss was strongly negatively associated with IQ of 7-year-old children^(50)^. In one of the studies in our review, hearing thresholds were also negatively associated with mental performance; children in the highest tertile of hearing thresholds performed significantly worse on the total mental test battery than children in the lowest tertile^(32)^. The interrelationship between hearing impairment and cognitive outcomes in relation to iodine status should be investigated in future studies.

Most studies in our review are limited by the fact that the hearing outcomes were based on hearing thresholds measured by air-conduction pure-tone audiometry. These should not be used as the sole measure of auditory development^(18)^ because they do not provide information on whether hearing differences are conductive (indicating problems with the outer and/or middle ear and mainly temporary) or sensorineural (indicating problems with the cochlea or beyond and permanent). They also do not assess auditory processing and individuals with hearing thresholds in the normal range might have difficulties in processing auditory information^(51)^. Therefore, the effect of iodine on these other aspects of hearing remains unknown.

### Limitations and conclusions

We included articles in the English language only; thus, we might have omitted relevant studies in other languages. The inclusion of all types of study design, including case-reports, is not optimal; however, considering the relatively unexplored nature of the topic, we believe this review provides a comprehensive account of the available evidence. It is important to note that two of the studies in our review also included adults (≥ 18 years)^(27,31)^; however, since the majority of participants in these studies were children and it was not possible to separate the data by age, we did not exclude these studies as we considered that they contribute to the overall evidence in children. The use of inconsistent measures of iodine intake/status (e.g. mean/median UIC or iodine-to-creatinine ratio, Tg, thyroid volume) across the included studies limited comparability between studies. The lack of a meta-analysis is a limitation; however, this could not be performed due to the scarcity of comparable and good-quality studies.

In conclusion, the evidence on the association between suboptimal iodine status and child hearing is based on few and mostly poor-quality studies; there is a lack of RCTs and interventions, with mostly observational evidence from cross-sectional studies. Most studies are in children, with limited evidence in pregnancy. The critical thyroid-hormone-dependent auditory development occurs predominantly *in utero* and therefore more maternal studies are needed. More observational evidence could be gathered from good-quality cohort studies that assess the association between maternal iodine status and child hearing. This would add to the evidence base and inform the design of future RCTs of iodine supplementation in pregnancy that might consider including an assessment of child hearing as one of the outcomes.

## Supplementary material

For supplementary material accompanying this paper visit https://doi.org/10.1017/S0007114522001441. click here to view supplementary material
