# Supplementary material for: Iodine status during child development and hearing ability: a systematic review
Source: Br J Nutr. 2022 May 10;129(5):795–812. doi: 10.1017/S0007114522001441 (PMC9975783; doi:10.1017/S0007114522001441)
Supplement: Supplementary file 1 [file S0007114522001441sup001.docx]

**Online Supplementary Material**

**Supplementary Methods.** Search terms for PubMed and Embase databases

**PubMed search code:**

(((iodine OR iodide OR iodate OR iodis* OR iodiz*) AND (thyroid OR thyroid function OR thyroid hormone) AND (hearing OR auditory OR deaf OR cochlea)) AND (Humans[MeSH] AND English [lang])) NOT (Covid OR povidone or radioactive)

**Embase search code:**

('iodine'/exp OR iodine OR 'iodide'/exp OR iodide OR 'iodate'/exp OR iodate OR iodis* OR iodiz*) AND ('thyroid'/exp OR thyroid OR 'thyroid function'/exp OR 'thyroid function' OR (('thyroid'/exp OR thyroid) AND ('function'/exp OR function)) OR 'thyroid hormone'/exp OR 'thyroid hormone' OR (('thyroid'/exp OR thyroid) AND ('hormone'/exp OR hormone))) AND ('hearing'/exp OR hearing OR auditory OR 'deaf'/exp OR deaf OR 'cochlea'/exp OR cochlea) NOT ('covid'/exp OR covid OR 'povidone'/exp OR povidone OR radioactive) AND [humans]/lim AND [english]/lim

**Supplementary Table S1.** Risk of bias in the included RCTs using the Cochrane RoB 2 tool*

|  |  | **Risk-of-bias judgment in RCTs** | | |
| --- | --- | --- | --- | --- |
|  |  | Gowachirapant *et al.* 2017 † |  | van den Briel  *et al.* 2001 ‡ |
| **Bias domain** |  |  |  |  |
| Bias arising from the randomisation process |  | Low |  | Some concerns |
| Bias due to deviations from intended interventions |  | Low |  | Some concerns |
| Bias due to missing outcome data |  | Low |  | Low |
| Bias in measurement of the outcome |  | Low |  | Low |
| Bias in selection of the reported result |  | Low |  | High |
| **Overall bias** |  | **Low** |  | **High** |

RCTs, randomised controlled trials; RoB 2, version 2 of the Cochrane risk-of-bias tool

* Sterne JAC, Savović J, Page MJ, et al. (2019) RoB 2: A revised tool for assessing risk of bias in randomised trials. *BMJ* 366.

† Gowachirapant S, Jaiswal N, Melse-Boonstra A, et al. (2017) Effect of iodine supplementation in pregnant women on child neurodevelopment: a randomised, double-blind, placebo-controlled trial. *Lancet Diabetes Endocrinol.* 5, 853–863.

‡ van den Briel T, West CE, Hautvast JGAJ, et al. (2001) Mild iodine deficiency is associated with elevated hearing thresholds in children in Benin. *Eur. J. Clin. Nutr.* 55, 763–768.

**Supplementary Table S2.** Risk of bias in the included non-randomised studies of interventions using the ROBINS-I tool*

|  | **Risk-of-bias judgment in non-randomised studies of interventions** | | |
| --- | --- | --- | --- |
|  | Azizi  *et al.* 2005 † |  | Wang and Yang, 1985 ‡ |
| **Bias domain** |  |  |  |
| Bias due to confounding | Serious |  | Serious |
| Bias in selection of participants into the study | Serious |  | Serious |
| Bias in classification of interventions | Serious |  | Moderate |
| Bias due to deviations from intended intervention | No information |  | No information |
| Bias due to missing data | Low |  | Low |
| Bias in measurement of outcomes | Moderate |  | Moderate |
| Bias in selection of the reported result | Moderate |  | Serious |
| **Overall bias** | **Serious** |  | **Serious** |

ROBINS-I, risk of bias in non-randomised studies - of interventions

* Sterne JA, Hernán MA, Reeves BC, et al. (2016) ROBINS-I: A tool for assessing risk of bias in non-randomised studies of interventions. *BMJ* 355.

† Azizi F, Mirmiran P, Hedayati M, et al. (2005) Effect of 10 yr of the iodine supplementation on the hearing threshold of iodine deficient schoolchildren. *J. Endocrinol. Invest.* 28, 595–598.

‡ Wang Y-Y & Yang S-H (1985) Improvement in hearing among otherwise normal schoolchildren in iodine-deficient areas of Guizhou, China, following use of iodized salt. *Lancet* 326, 518–520

**Supplementary Table S3.** Quality assessment of the included prospective cohort studies and cross-sectional studies using the Newcastle-Ottawa scale*

| **Study**  (first author, year) | **Selection**  (max 4 stars) | **Comparability** (max 2 stars) | **Outcome assessment** (max 3 stars) | **Overall quality judgement** † |
| --- | --- | --- | --- | --- |
| **Prospective cohort studies (n=1)** | | | | |
| Hynes, 2017 ‡ | ★★★ | ★★ | ★★ | Good |
| **Cross-sectional studies (n=6)** | | | | |
| Goslings, 1975 § | ★★ |  |  | Poor |
|  |  |  |  |  |
| Todd, 1988 \|\| | ★★ | ★ |  | Poor |
|  |  |  |  |  |
| Valeix, 1994 ¶ | ★★★ |  | ★★ | Poor |
|  |  |  |  |  |
| Azizi, 1995 ** | ★★ | ★ | ★★ | Fair |
|  |  |  |  |  |
| Soriguer, 2000 †† | ★★★ | ★ | ★ | Poor |
|  |  |  |  |  |
| Scinicariello and Buser, 2018 ‡‡ | ★★★★ | ★★ | ★★★ | Good |

* Wells GA, Shea B, O’Connell D, et al. *The Newcastle-Ottawa Scale (NOS) for assessing the quality of nonrandomised studies in meta-analyses*. Ottawa: Ottawa Hospital Research Institute Available at: http://www.ohri.ca/programs/clinical_epidemiology/oxford.asp [Accessed April 27, 2019]

† Overall quality grading: **1) good =** 3 or 4 stars in selection domain AND 1 or 2 stars in comparability domain AND 2 or 3 stars in outcome domain; **2) fair =** 2 stars in selection domain AND 1 or 2 stars in comparability domain AND 2 or 3 stars in outcome domain; and **3) poor =** 0 or 1 star in selection domain OR 0 stars in comparability domain OR 0 or 1 stars in outcome domain.

‡ Hynes K, Otahal P, Burgess J, et al. (2017) Reduced Educational Outcomes Persist into Adolescence Following Mild Iodine Deficiency in Utero, Despite Adequacy in Childhood: 15-Year Follow-Up of the Gestational Iodine Cohort Investigating Auditory Processing Speed and Working Memory. *Nutrients* 9, 1354.

§ Goslings BM, Djokomoeljanto R, Hoedijono R, et al. (1975) Studies on hearing loss in a community with endemic cretinism in central Java, Indonesia. *Acta Endocrinol. (Copenh).* 78, 705–713.

|| Todd C, Sanders D & Chimanyiwa T (1988) Hearing in primary school children in an iodine-deficient population in Chinamhora, Zimbabwe. *Trop. Geogr. Med.* 40, 223–225.

¶ Valeix P, Preziosi P, Rossignol C, et al. (1994) Relationship between urinary iodine concentration and hearing capacity in children. *Eur. J. Clin. Nutr.* 48, 54–59.

** Azizi F, Kalani H, Kimiagar M, et al. (1995) Physical, neuromotor and intellectual impairment in non-cretinous schoolchildren with iodine deficiency. *Int. J. Vitam. Nutr. Res.* 65, 199–205.

†† Soriguer F, Millón MC, Muñoz R, et al. (2000) The auditory threshold in a school-age population is related to iodine intake and thyroid function. *Thyroid* 10, 991–999.

‡‡ Scinicariello F & Buser MC (2018) Association of iodine deficiency with hearing impairment in US adolescents aged 12 to 19 years: analysis of NHANES 2007-2010 data. *JAMA Otolaryngol. - Head Neck Surg.* 144, 644–645.
